# Mind your language: the importance of english language skills in an International Medical Programme (IMP)

**DOI:** 10.1186/s12909-022-03481-w

**Published:** 2022-05-26

**Authors:** Sharon Min Hui Chan, Norul Hidayah Mamat, Vishna Devi Nadarajah

**Affiliations:** 1 Centre for Pre University Studies, International Medical University, Kuala Lumpur, Malaysia; 2 Centre for Education, International Medical University, No 126 Jalan Jalil Perkasa 19, Bukit Jalil , 57000 Kuala Lumpur, Malaysia; 3 Education and Institutional Development Office, International Medical University, Kuala Lumpur, Malaysia

**Keywords:** English Language skills, Medical education, Medical students, International medical programmes

## Abstract

**Background:**

Language proficiency is crucial for doctors as they communicate with patients, peers and other healthcare professionals. Although proficiency in English is part of admission requirements, there is a gap in the knowledge of medical students’ perception of factors enhancing English language (EL) skills during training in international medical programmes (IMPs). The gap prevents educators and policy makers from helping students who struggle with communication skills during medical training. This study therefore explores the importance of English language skills from medical students’ perspectives.

**Methods:**

Six focus group interviews with 24 medical students were conducted in an IMP. Data were analysed using Braun and Clarke’s framework of thematic analysis.

**Results:**

Results established three themes that constitute the importance of EL skills, namely the use of EL in medical training and practice, influence of university culture in EL mastery and individual EL proficiency as perceived by medical students.

**Conclusions:**

Findings of this study demonstrate how students perceived the importance of EL skills as a professional and social requirement during medical training and for future practice. It also informs that setting English language admission pre requisites needs to be complemented with opportunities to practice context specific communication skills. Thus, international medical programmes should embed diverse and inclusive strategies to support and develop medical students’ English language skills.

**Supplementary Information:**

The online version contains supplementary material available at 10.1186/s12909-022-03481-w.

## Background

### English language and medical education

The importance of English language (EL) skills in medical education is demonstrated when students from multilingual backgrounds enrol into international medical programmes (IMPs) [1]. The past three decades have seen an increasing number of universities with global perspectives of health professions education offering IMPs [2]. With IMPs, the curriculum, teaching, learning and assessment activities are conducted in English [3]. EL serves as prerequisite for enrolment into IMPs which include assessments such as International English Language Testing System (IELTS) or its equivalent, Test of English as a Foreign Language (TOEFL). Originally, EL assessments were meant to assess the language ability of candidates who need to study or work where English is used as the language of communication. Nonetheless, higher education institutions in Anglophone countries seem to have broadened the use of these assessments to gauge students’ academic performance [4].

English is an international language of medicine. It is also expanding as an international communication and educational tool [5]. The literature emphasises the importance of communication skills among medical practitioners as “effective doctor-patient communication is a crucial aspect of quality patient care” [6]. However it is scarce in relation to the perception of medical students towards factors enhancing English language and communication skills. This particularly applies in multilingual environments and IMPs [7]. Studies on medical students’ English language needs in Taiwanese higher education institutions were mostly concerning English language teaching [8]. The study highlighted that although students value English proficiency in their training as medical doctors, universities failed to satisfy students’ expectations and facilitate attainment of language proficiency [8]. In these environments, students are faced with double cognitive task [9]; studying English as the medium of their chosen studies while trying to improve their English proficiencies [10]. Similarly, in a study from a Gulf University, students acknowledged and identified weakness in English language as a hindrance to their communication and information handling skills [10].

This context highlights the need for studies to identify factors that enable enhancement of English language skills from the perspectives of medical students. The current knowledge gap prevents educators and policy makers from helping students who struggle with communication skills during medical training [11]. Findings from this study will be useful in determining learning and support strategies for students in both multilingual settings and IMPs.

### Vygotsky’s sociocultural theory

Given the context which relates to the importance of the English language and the gaps in medical education literature on factors enhancing EL skills, the theoretical framework adopted for this study is the sociocultural theory by Vygotsky (1978). This framework posits that an individual’s learning is linked to cultural, institutional and historical contexts [12]. The four core tenets of Vygotsky’s sociocultural theory which are (i) learning precedes development, (ii) language is the main vehicle (tool) of thought, (iii) mediation is central to learning and (iv) social interaction is the basis of learning and development [13]. The tenets can be adapted to the study learning environment ranging from curricula outcomes to teaching and learning methods through social interactions. The tenets which initially focused on children’s learning have been extended to include adult learning. The application of this theory to adult learning guides in recognizing the patterns of learning present in the learning environment [14]. Henceforth, the use of this theory is preferred as it encapsulates learning as a continuous process of internalization during training, in which development of EL skills and knowledge are transformed and intertwined between the social and cognitive planes.

When medical students participate in conversations and activities in a multicultural group, they will be able to connect with their peers, and assimilate the experience, which includes use of EL in medical training [11]. Therefore, students engage in a wide range of joint activities and subsequently, acquire new strategies and knowledge of the culture in which they are placed [15].

In summary, the gaps in literature and the broad contextual needs and similarities of IMPs led to this study exploring medical students’ perspectives on the importance of EL skills in medical training.

## Methods

### Sampling context

A total of 24 medical students from Year 1, 2 and 3 of a five-year IMP [2] participated in six focus group interviews. The students have fulfilled a minimum IELTS requirement of an overall band score between 6.5 to 7.0 for all sections (reading, writing, speaking and listening) upon entry to the IMP. We used maximum diverse sampling method to recruit participants based on the inclusion criteria of medical students who were in Year 1 to Year 3. The selection of participants from Year 1 to 3 provided the range of pre-clinical and clinical phases to ensure that data could be obtained from participants at different stages of the IMP.

### Data collection

 Informed consent was obtained from participants prior to each focus group interview. The interview was audio recorded to facilitate transcription. The interview questions were categorised in three areas. The first area, English language usage in medical education, consisted of two questions; *‘How is English language used in medical studies?’* and *‘In what ways did you see the usage of English Language in school compared to university?*’. In the second area, the impact of English language proficiency on medical students, the interview question was; *‘Can you explain instances where learning was impeded by English language?’*. Likewise, in the third area, the influence of university culture in English language mastery, the interview question was; *‘In what way has the university learning environment enhanced your mastery of English language?’*. Each question was probed to gain in depth perspective of the importance of English language skills. At the end of the interview, participants were given the opportunity to share additional perspectives and contribute further. The interviews were transcribed and coded to identify similarities and differences of perspectives. The saturation was deemed to be achieved when similar and redundant perspectives shared by the participants and no new information were obtained across the focus groups.

### Data analysis 

The data were analysed using the Braun and Clarke’s framework of thematic analysis [16]. The framework includes six steps: 1) Data familiarization, 2) Identification of codes, 3) Finding themes, 4) Reviewing of themes, 5) Definition and naming of themes and 6) Report production. It provides a clear and usable framework, distinguished at two levels; semantic and latent. At the semantic level, the explicit or surface meanings of the data, based on surfaced themes are analysed, while at the latent level, the analysis looks beyond what was said during the focus group discussions, giving it more depth in analysis [16]. In Step 1, the data from the transcription was repeatedly read to familiarize transcript and identify the codes. Samples of codes from transcription data are shown in Additional file 1: Table S1. In Step 2, the coding process started with initial codes generated on *students’ perception of English language*, *the availability of university support on mastery of English language* and *the impact of English language on medical students,* as shown in Additional file 1: Table S1. In Step 3, the subsequent coding process involved examinining the identified codes and narrowed it into more specific codes. The codes identified in Step 1 and Step 2 were further reviewed and led to emergence of preliminary themes such as *‘English language as a learning tool’*, *‘university support in English language mastery’* and *‘English language for academic progression’*. In Step 4, the preliminary themes identified through data coding that represented the context of the importance of English language skills were reviewed and refined. In Step 5, a total of three themes were defined and established. Finally in Step 6, results based on the established themes were finalised.

### Research team and reflexivity

The first research team member is a language teacher who has a background in linguistics. The second member is trained in educational psychology and teaches in the Health Professions Education Programme. The third team member is a biochemist and medical educationist by training. Throughout the study, the team was actively involved in the research process while at the same time aware of subjectivity due to their different backgrounds.

## Results

A total of three themes were established, namely, *‘EL usage in medical training and practice’*, **‘the influence of university culture and environment in EL mastery’** and *‘individual EL proficiency’*.

## Theme 1: English language usage in medical training and practice

In the medical programme, learning activities include lectures, problem-based learning sessions, clinical skills and simulation sessions, hospital and bedside teaching. It is evident that students perceived EL as a learning tool in these activities as they cited that activities were in English.

“If we're in class, sure, English only speaking, if we're at Clinical Skills & Simulation Centre sure, English only speaking.”

Furthermore, students also perceived EL to be an important learning tool as medical learning resources were mainly in English.

“Reference or the books that we use are mostly used in English. So, English is very important actually in our studies of our medical programme”.

English language is perceived to be equally important as a professional language. Medical students communicate in English with patients, peers and lecturers and participate in external events such as community support, conferences and extra curricular activities (ECAs). Students shared that speaking in English made them appear more professional, particularly when they represented the university in external events.

*“It's also something to do with the image because like if we do if we are representing IMU and going to a different public university in Malaysia for example. So if we since we are carrying the name of IMU, if we converse in English, I would say that it will bring a better image to IMU”.*

Medical students communicated with peers formally and informally in the learning setting and during social interactions. Furthermore, when students were from multilingual backgrounds and nationalities, English language was also the main communication tool.

*“We have people from various cultural backgrounds and different countries, even we have international students here. So, it's logical if we speak in English and we converse with each other in English since that that will make the international students feel more comfortable as they will be able to understand what we're talking.”*

## Theme 2: The influence of university culture and environment on EL mastery

As students were multilingual with varied cultural backgrounds and nationalities, it was necessary to explore university support in mastering the English language. Students felt that the university learning environment with English as a medium of instruction and extra curricular activities helped in strengthening their command of English. There was obvious improvement in English language proficiency for peers who had initially struggled with English language after the first semester of the medical programme.

*“But what I noticed from them is that after the six months of our semester one their English skills like improve tremendously. And when we asked the person about it, what they said was that it was the talking with all the peers was the one that helped them.”*

To support improvement in EL communication skills, students mentioned that lecturers equally encouraged them to communicate in English.

“Our lecturer actually encouraged him to speak English more.”

Nonetheless some students faced difficulties learning and communicating in an English speaking environment.

“At first I was quite struggling to adapt with English speaking environment.”

Students from multilingual and multicultural backgrounds with lesser opportunities to communicate socially in English expressed that the English speaking environment came as a shock, as some of them did not have experience of speaking with different ethnicities or nationalities. Therefore, in order to improve their English proficiency and cope with learning, students mentioned that they took initiatives on their own and approached peers for support.

*“If they really don't know the language, they will come to us and ask us what does it mean… We have to translate it to them, either using their own language, or we use really simple terms to explain it.”*

Besides that, students watched and listened to English reading and entertainment materials, online learning tools and registered for English classes outside the university.“I tried to improve myself with a lot of things like, I watch English movies, I listened to English songs. And of course, I tried to gain the courage to speak in English with my friends.”

## Theme 3 – Individual EL proficiency

Students felt that limitations in EL impacted their learning as they needed more time and effort to comprehend the resources.

*“They might actually be speed lagging behind learning like learning the syllabus, I mean, they have to be/ they have to put like extra effort to learn the same thing that we learned/ we have to learn because maybe it's not in the language that they are comfortable in.”*

Furthermore, assessments such as assignments, presentations, reports and the Objective Structured Clinical Examinations were carried out in English. Hence, having poor command of English had an impact on their assessments when examiners were not clear about what was presented.

*“If you are not so proficient in the language, you will have difficulty trying to structure your points according to paragraphs and making the flow look nice. And because of the inability to do that, then the lecturer, the person marking the paper might feel like, this person is all over the place, he doesn't really know what he's writing about.”*

Lack of English proficiency impeded students’ day to day learning. Some students were afraid to ask their lecturers when unsure of the content taught or participated less in problem based learning discussions.

“It’s hard when you are among with your peers because you need to let them to understand and also the facilitator to understand. Some they don't know certain my language.”

Students also perceived that proficiency in EL had an impact on patient communication. Lack of English proficiency may cause the interactions to sound rude, awkward and lead to misunderstanding and errors in instructions given to patients. For sensitive issues such as sexual history, medical students explained that proficiency in English is needed when exploring details without causing patients’ uneasiness. Most importantly, they felt proficiency helped gain a patient’s confidence when discussing such matters.

*“I think communication with patients, that will also be a problem because some people I heard my friends say, they directly translate it from Mandarin to English. And then sometimes, it might sound wrong, it might sound rude in English.”*

During their studies, students were involved in medical research, met with visiting professors from other universities, attended conferences and received training at health clinics and wrote case reports. These encounters, which were mainly in English, contributed to the personal and professional development of a future doctor. Students explained that EL is important to review literature, conduct and communicate research. This resulted in opportunities to interact professionally in academia or healthcare at the workplace or when collaborating with international colleagues.

*“…eventually when you go out into the working world, when you do literature reviews, when you review other people's research, when you do your own research, eventually you're going to have to reach that level of like academic, that sort of academic standard that is required of the community when they do research projects, and you do literature reviews.”*

*“For me usage of English might not be very important in a community level, but for professional level like from colleagues to colleagues, we still need to use English or professionalism and to discuss anything about medical, I think it's very important to speak in English.”*

## Discussion

The aim of this study was to explore the importance of English language skills in an IMP from medical students’ perspectives. Evidence obtained from the perspectives resonates with Vygotsky’s sociocultural theory, its tenets and model that learning is a continuous process and involves multiple factors in promoting the language usage and its importance within the context of medical education and multilingual settings.

The bidirectional elements of culture, thought and language showed that the importance of EL skills are multi faceted, aiding in developing contextual social and cognitive skills in multilingual healthcare settings. This is demonstrated through the emphasis on usage of English during medical training and practice, the influence of university culture and environment on EL mastery and students’ individual EL proficiency.

Medical students identified that they learnt EL skills through participation in facilitated teaching and learning activities such as problem-based learning sessions and simulation and clinical skills training. These learning activities provide an opportunity for students to communicate with peers, simulated and real patients. Vygotsky puts forward the concept of Zone Proximal Development (ZPD) in which a learner’s level of potential development is determined through adult guidance or collaborative work with more capable peers [17]. Through guided participation, learning and development take place in a social context between learner and teacher [12] allowing medical students to enhance their EL skills. Evidence showed that lecturers emphasised the importance of communication skills and linked it to accuracy in the EL vocabulary.

Besides that, students acknowledged that they improved their language skills through peer interaction during extra curricular activities. Vygotsky emphasized the dominant effect of social experience on human development was that thoughts developed through social interaction will be internalized through the cultural context in which the learner is placed [18]. Therefore, students will develop thought and knowledge within the community of practice where learning in a second language context takes place [19]. To this effect, evidence showed that multilingual students who acquired English language skills experienced the assimilation process through peer learning. This correlates with a study conducted among university students from a multicultural background in Canada and Russia on the perceived impact of ECA on foreign language learning. Participants of the study indicated that they had more opportunities to practice speaking in English language, learnt more, strengthened their comprehension and developed communication skills when participating in extra curricular activities [20].

Additionally, students observed that in both learning and healthcare environments, EL is used in a professional setting amongst healthcare professionals and for patient care. Vygotsky proposed that mediation (a tool) is often used by learners to resolve a problem or achieve a target, and hence language is considered a significant tool for learners to develop the knowledge that they require [17]. In this context, medical students reminded educators that the EL, as a communication tool, was crucial for person-centred care whereby communication skills is crucial for patient engagement and safety of care [21]. This importance as a communication tool is expanded to peers and other health professionals and helps them develop personally and professionally, opening opportunities to further their medical education abroad, specialise or conduct research.

However, students also identified the challenges faced in acquiring EL skills especially in a multilingual environment and the subsequent impact that may occur from the lack of it. Therefore, they employed various strategies such as getting peer assistance, enrolling in external language courses to cope with these challenges. Additionally, efforts can be made in the curriculum to support medical students with English learning. At our institution, this was done through the introduction of English Literature and Medicine module as an optional elective for medical students. Whilst learning takes places informally through observations of doctors’ and patients’ interactions and discussions of patients’ cases, these interactions are often neglected in clinical reports [22]. Hence in fostering medical students’ doctor-patient communication, the module was designed to awaken students’ emotions and awareness toward empathetic behaviours through English literary texts. More recently another elective module for stories and perspective taking was introduced to understand patient care. In a study to explore the effectiveness of this newly introduced module for dental students, the findings showed that it facilitates empathy in students, stimulates their self-awareness and motivates students to be perceptive communicators [23].

The study findings highlighted the need to continously make available EL enhancement programmes and support structures for medical students. Whilst IMPs already have requirements of English competence prior to entry [24], generic tools such as IELTS or TOEFL, may be insufficient for some students to navigate through medical training. Even at our institution, where EL support is given for students identified through mentor feedback, more can still be done to support students. There are students, as indicated in this study, who would like to gain more social and academic experience in EL to thrive in medical school. One initiative that has been gaining ground is the introduction of humanities to enhance inter disciplinary collaboration, patient communication and care [22]. A study conducted among medical and healthcare students in Taiwan concluded that narrative medicine has positive effects on the students whereby it helped with interdisciplinary collaboration and enabled them to listen to illness narratives from multiple perspectives [25]. Recommendations would be to invest and increase access to these types of programmes, diversify the narratives to be inclusive of contemporary culture and arts, and to intergrate these programmes with clinically related modules in the medical curriculum, rather than offer them as stand alone modules. Concordantly, education of medical and nursing students is suggested to include interdisciplinary experiences in humanities and its’ relevance to the practice of healthcare providers [26].

This study was limited by the perceptions of medical students of an IMP in a single institution. Furthermore, as it was challenging to encourage students to participate in focus groups, students who participated in the focus groups may not fully represent all IMP students. With the increasing globalisation of higher education and English as the common language of higher education, medical students of multi demographic settings may have similar or different perception of the importance of EL skills.

For future studies, the scope of this study can be broadened to include senior, near graduation or junior doctors as they may offer a closer to workplace perspective. Besides that, medical students' perspective in bilingual and monolingual settings can also be investigated and compared to identify similarities and differences in medical students' experiences.

In conclusion, various factors come into play during the students’ journey to enhance their EL proficiency. As seen in the results, for the medical students, university culture and institutional support both formally and informally matters as much as students’ self-initiatives and peer support. The context of medical training which includes patient communication, peer interactions and future practice in healthcare also increased the awareness and interest to be proficient in English amongst medical students. Hence, it is imperative that IMPs continue to invest in and broaden approaches both within the formal curriculum and extracurricular activities to support and develop medical students' EL skills.

## Supplementary Information

**Additional file 1: Table S1.** Sample of Codes.

## Data Availability

Data were obtained through focus group interviews, provided in Additional file 1: Table S1 and the datasets used and/or analyzed during the current study are available from the corresponding author upon reasonable request.
