# Supplementary material for: Mind your language: the importance of english language skills in an International Medical Programme (IMP)
Source: BMC Med Educ. 2022 May 26;22:405. doi: 10.1186/s12909-022-03481-w (PMC9134667; doi:10.1186/s12909-022-03481-w)
Supplement: Supplementary file 1 — Additional file 1: Table S1. Sample of Codes. [file 12909_2022_3481_MOESM1_ESM.pdf]

Additional file 1: Table S1. Sample of Codes

THEMES	Theme 1: English language usage in medical training and practice			Theme 2: The influence of university culture and environment on EL mastery		Theme 3: Individual EL proficiency		
CODES	***Learning tool***	***Professional language***	***Communication tool***	***University support***	***Self-initiative and peer support***	***Academic progression***	***Patient communication***	***Personal and professional development***
PHRASES	For understanding	Used in workplace setting with patients and doctors	Communicate with foreign students	Not corrected on language proficiency	Peer support	In describing findings	Usage of simpler words in clinical setting for patients' understanding	Formal discussions
	Ensure understanding	Communication with lecturers on emails	For communication with ppl out of imu, at interviews and other matters	Prefer to be corrected	Peer support	Challenges learning medical terms	Simplify english to ensure patients understand message conveyed	Among health professionals to discuss medical related matters
	Medium of discussion	Among health professionals to discuss medical related matters	'safe' language for discussions	Punctuation corrected by lecturer in report	Learn vocabulary in context automatically	More effort needed to learn because syllabus is taught in english		Hindrance to learning
	Language for discussion	To converse with patients and superior	Reduce level of english so the other person can understand	Encourage ment from lecturer	Reduce level of english to accommodate peers in class	Requires more time to learn syllabus		
	Primary language			Grammar errors not corrected on	Online translating tools	Hinders presentation		
	Content over accuracy			Being corrected on vocabulary				
	Curriculum over language			Unsure of university support				
	Common language			Not corrected on language proficiency				
				Prefer to be corrected				
				Through participatio n in university activities				
